# Opsonization and timing as key determinants of MBTA immunotherapy efficacy in pancreatic adenocarcinoma and recurrence treatment

**DOI:** 10.1080/15384047.2026.2683273

**Published:** 2026-06-19

**Authors:** Andrea Frejlachova, Radka Lencova, Ondrej Uher, Katerina Hadrava Vanova, Klara Martinkova, Monika Cizkova, David Vetvicka, Helena Langhansova, Jan Kopecky, Karel Pacak, Jan Zenka

**Affiliations:** a Department of Medical Biology, Faculty of Science, University of South Bohemia, Ceske Budejovice, Czech Republic; b BioCanim a.s., Prague, Czech Republic; c Section on Medical Neuroendocrinology, Eunice Kennedy Shriver National Institute of Child Health and Human Development, National Institutes of Health, Bethesda, MD, USA; d Institute of Biophysics and Informatics, First Faculty of Medicine, Charles University, Prague, Czech Republic; e Center of Adrenal Endocrine Tumors, AKESO, Prague, Czech Republic; f Faculty of Medicine, Palacky University, Olomouc, Czech Republic; g Faculty of Medicine, 5thDepartment of Medicine, Commenius University, Bratislava, Slovakia; h TumorShot, Prague, Czech Republic

**Keywords:** Pancreatic adenocarcinoma, immunotherapy, TLR agonists, anti-CD40, phagocytosis, recurrence

## Abstract

**Background:**

Pancreatic adenocarcinoma is a highly aggressive cancer with very limited treatment options. This study aimed to optimize the efficacy of a previously developed tumor immunotherapy for the treatment of this disease and its recurrences.

**Methods:**

Mouse models of pancreatic and colon adenocarcinoma were established using Panc02 and MC38 cells, respectively. Tumors were treated by intratumoral administration of MBTA, a formulation containing resiquimod, poly(I:C), LTA, anti-CD40 antibody, and mannan-BAM (a phagocytosis-stimulating mannan anchored to the tumor cell membrane via a biocompatible membrane anchor, BAM). Multiple variants of the therapy were tested, differing in composition and timing, including treatment of recurrences.

**Results:**

Intratumoral MBTA immunotherapy administered using an optimized 5 × 2 schedule resulted in an 87.5% survival rate in mice bearing subcutaneous Panc02 tumors. MBTA immunotherapy also effectively treated spontaneous local Panc02 recurrences that developed in a small subset of mice. High efficacy of MBTA was further confirmed in a murine model of colon adenocarcinoma.

**Conclusion:**

These findings suggest that MBTA is a promising therapeutic approach for primary and recurrent pancreatic adenocarcinoma, with potential for broader clinical application.

## Article highlights

- The combination of TLR signaling and phagocytic receptor ligands is crucial for cancer immunotherapy based on mannan-BAM, TLR ligands, and anti-CD40 antibody (MBTA).
- MBTA immunotherapy reduces the risk of disease recurrence and prolongs survival.
- The timing of immunotherapeutic application significantly influences the effectiveness of immunotherapy. The 5x2 treatment regimen proved to be optimal (MBTA administered on days 0, 1, 7, 8, 14, 15, 21, 22, 28, and 29).

## 1. Introduction

Cancer immunotherapy has become an attractive therapeutic approach in recent years, leading to the development of new treatment strategies. However, only a minority of patients respond effectively, underscoring the limitations that remain.^1^ We hypothesized that successful elimination of cancer cells would require interplay between innate and adaptive immunity with subsequent development of immunological memory. In tumors, however, the function of the immune system is severely limited. Tumor cells often downregulate or mask antigens that would normally trigger immune recognition, and they also create an immunosuppressive microenvironment preventing effective tumor elimination. To overcome these barriers, our concept is based on stimulation of phagocytosis activating innate immune cells, and subsequently initiating and amplifying adaptive immune responses and promoting the establishment of long-term immunological memory.

**Figure 1.**
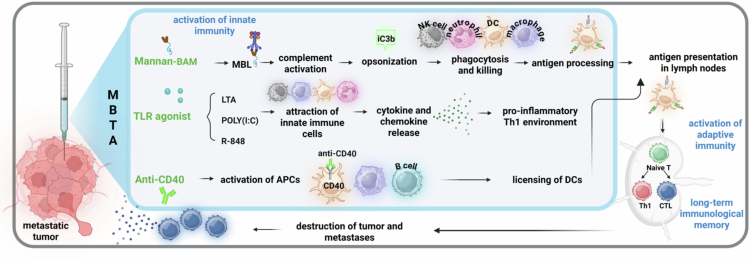
Mechanism of MBTA immunotherapy. After the injection of MBTA. immunotherapeutic mixture into the tumor, mannan is anchored into the lipid bilayer of tumor cells via a biocompatible anchor for the cell membrane (BAM). Subsequently, mannan on the cell surface is recognized by mannose-binding lectin (MBL), which leads to the activation of the complement system through the lectin pathway. This process initiates the formation of the membrane attack complex (MAC) and opsonization of tumor cells by iC3b molecules. The TLR ligands contained in the MBTA mixture attract innate immune cells (neutrophils, macrophages, natural killer cells (NK cells), dendritic cells (DCs)) into the tumor microenvironment. The iC3b is recognized by the C3 receptor on the surface of innate immune cells and they then use their effector mechanisms to eliminate such opsonized tumor cells. The activation of TLR receptors on innate immune cells triggers signaling cascades, which leads to the production of pro-inflammatory cytokines, such as IL-1β, IL-6, IL-12, and TNF-*α*, thereby inducing a proinflammatory Th1 environment. The anti-CD40 antibody binds to receptors on antigen-presenting cells (APCs), such as macrophages and dendritic cells, leading to their activation. Subsequently, these APCs present tumor antigens to T lymphocytes in the lymph nodes, thereby inducing T cell activation and eliminating rest of primary tumors and metastases.

Building on this rationale, we introduced and optimized MBT immunotherapy, which combines toll-like receptor (TLR) ligands – resiquimod (R-848), poly(I:C), and lipoteichoic acid (LTA) with the phagocytosis-stimulating ligand – mannan. The best results were obtained when mannan was anchored to the tumor cell membrane using a biocompatible anchor for cell membranes (BAM).^2^ ^,^ ^3^ Mannan-BAM is subsequently recognized by mannan-binding lectin, which activates the complement cascade and leads to iC3b opsonization of tumor cells.^4^ TLR ligands induce robust infiltration of immune cells into the tumor microenvironment, promote the establishment of a Th1-type immune response, and create a favorable milieu for antigen presentation to T lymphocytes.^5^ In previous studies, this combination (MBT immunotherapy) eradicated B16-F10 murine melanoma in 83% of treated mice.^6^ Furthermore, in more aggressive murine cancer models, such as pancreatic adenocarcinoma (Panc02 cells) and pheochromocytoma (MTT cells), MBT immunotherapy similarly reduced tumor growth; however, survival after immunotherapy was limited. To improve survival outcomes, MBT immunotherapy was later on successfully combined with an agonistic anti-CD40 antibody, resulting in MBTA immunotherapy.^6^ ^,^ ^7^ The overall mechanism of MBTA immunotherapy is illustrated in Figure 1. A comprehensive description of the ongoing processes is provided by Uher et al.^4^

The central principle of MBTA immunotherapy is proposed to rely on the functional cooperation between signaling molecules (TLR agonizts and anti-CD40) and the stimulation of phagocytosis (mannan-BAM), based on previously published mechanistic studies.^4-6^ Given the extensive optimization of signaling pathways, it remained uncertain whether artificial opsonization of tumor cells with mannan-BAM is still required to achieve full therapeutic efficacy. In addition, questions regarding the enhancement of MBTA effectiveness, the treatment of spontaneous recurrences, and the optimization of timing and dosing schedules have not yet been resolved. To address these issues, we performed a series of experiments in murine models of pancreatic and colon adenocarcinoma, using the functional cooperation of signaling and phagocytic molecules, focusing on recurrences and prolongation of survival, the optimization of timing, and dosing of MBTA immunotherapy.

## 2. Results

### 2.1. Functional cooperation of signaling and phagocytic molecules during MBTA immunotherapy of murine pancreatic adenocarcinoma

**Figure 2.**
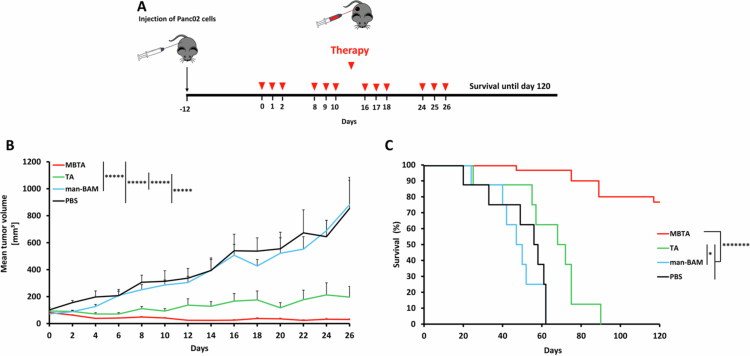
Comparison of the effect of MBTA, TA and mannan-BAM immunotherapy in Panc02 tumor-bearing mice with subsequent survival analysis. **(A)** C57BL/6JOlaHsd mice were subcutaneously injected with Panc02 cells in the right flank. After 12 d, mice were randomized into four groups: MBTA (TLR agonizts + anti-CD40 antibody + mannan-BAM, *n* = 30); TA (TLR agonizts + anti-CD40 antibody, *n* = 8); mannan-BAM alone (*n* = 8); and vehicle control (PBS, *n* = 8). All solutions were applied in pulse regimen (days 0, 1, 2, … 8, 9, 10, … 16, 17, 18, ... 24, 25, 26). **(B)** Tumor growth is shown as a growth curve. Statistical analysis was performed on AUC values by one-way ANOVA with Tukey's *post hoc* test (*****p < 0.0005). **(C)** The survival analysis is presented as a Kaplan–Meier curve, log-rank evaluation (*p < 0.05, ******* *p* < 0.00005).

The aim of this experiment was to evaluate the therapeutic efficacy of a mixture of TLR agonizts with anti-CD40 (TA), to compare it with the effect of the phagocytosis stimulator mannan-BAM and to assess the synergistic effect of combining TA with mannan-BAM (MBTA). The experimental timeline is shown in Figure 2A. The mean tumor volume at the start of the experiment was 82.7 ± 30.1 mm^3^. Interestingly, TA alone induced a significant reduction in tumor growth and prolonged survival (Figure 2B, C); however, this treatment did not lead to complete tumor eradication or cure (Figure 2C). By contrast, the addition of mannan-BAM was critical for achieving complete remission in the majority of mice (Figure 2C). These findings support the importance of combining signaling molecules with phagocytosis-stimulating components for achieving maximal therapeutic efficacy of this immunotherapy.

### 2.2. Immunotherapy approach for recurrences associated with prolongation of survival in murine pancreatic adenocarcinoma

**Figure 3.**
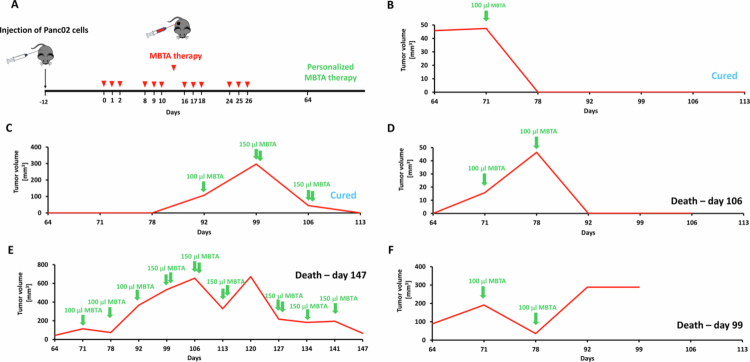
Personalized MBTA immunotherapy of tumor recurrences. **(A)** Twenty-seven C57BL/6 mice were subcutaneously injected with Panc02 cells in the right flank. After 12 d, mice were randomized and treated by intratumoral application of MBTA in pulse regimen (days 0, 1, 2, … 8, 9, 10, … 16, 17, 18, ... 24, 25, 26). **(B–F)** From day 64 onward, tumor recurrence was monitored and personalized MBTA immunotherapy was initiated in individual mice with the aim of achieving complete cures.

In previous experiments, MBTA immunotherapy successfully cured most mice with various tumors (melanoma, pancreatic adenocarcinoma, pheochromocytoma,^4-6^ ^,^ ^8^ however, achieving a 100% cure rate remains challenging, primarily due to tumor recurrence. We therefore sought to identify strategies to reduce or fully cure these relapses. Given the general effectiveness of MBTA immunotherapy and the infrequent occurrence of recurrences, subsequent experiments were performed on 27 mice. At eight weeks of age, mice were transplanted with Panc02 cells and treated with MBTA (Figure 3A). After completion of immunotherapy, tumor growth was continuously monitored. Tumor recurrence was observed in 5 mice on days 64–78 (18.5%). For these animals, we initiated personalized MBTA treatment according to tumor progression (Figure 3B–F). Complete remission was achieved in two mice (Figures 3B, 2C), one of which was notably cured with a single injection of MBTA (Figure 3B). In the remaining relapsed mice, survival was substantially prolonged: up to 106 d (Figure 3D), 147 days (Figure 3E; injected 3 × 100 and 10 × 150 μl of MBTA), and 99 d (injected 2 × 100 μl of MBTA (Figure 3F). In parallel, a control group of eight Panc02-bearing mice was treated with PBS, resulting in a mean survival of 50.13 ± 11.90 d.

In a subsequent experiment, this approach was repeated in 39 mice. Tumor recurrence was observed in 3 mice (7.7%) on day 50. In this case, MBTA immunotherapy was administered uniformly in a pulse regimen, consisting of intratumoral injection of 100 μl MBTA on days 56, 57, 58, 64, 65, 66, 72, 73, 74, 80, 81, and 82. The results were consistent with the previous study: one mouse achieved complete remission, while two showed markedly prolonged survival, succumbing on days 118 and 119. In parallel, a control group of eight Panc02-bearing mice treated with PBS exhibited a mean survival of 43.5 ± 9.15 d (data not shown).

### 2.3. Optimizing the timing and personalization of MBTA immunotherapy in murine pancreatic adenocarcinoma

**Figure 4.**
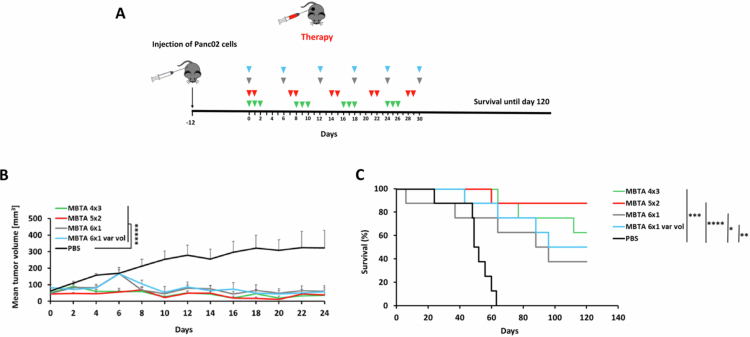
Optimization of MBTA timing and personalization of intratumoral immunotherapy. **(A)** C57BL/6 mice were subcutaneously injected with 4 × 10^5^ Panc02 cells in the right flank. After 12 d, mice were randomized into five groups (*n* = 8/group) and treated by intratumoral application of 50 µl MBTA according to the indicated regimens. **(B)** Tumor growth is shown as a growth curve. In the “MBTA 6 × 1 var vol” group, the MBTA volume was personalized to match tumor size (MBTA volume = 1/2 tumor volume). Statistical analysis was performed on AUC values using one-way ANOVA with Tukey's post hoc test (*****p < 0.0005). **(C)** The survival analysis is presented as a Kaplan–Meier curve, with log-rank test evaluation (*p < 0.05, **p < 0.01, ***p < 0.005, ****p < 0.001).

Immunotherapy of Panc02 pancreatic adenocarcinoma with MBTA was performed using different dosing schedules (Figure 4). The objective was to identify the most effective regimen with the fewest injections, thereby facilitating future clinical translation. Three schedules were tested: i/4 × 3 (four cycles of three injections; MBTA administered on days 0, 1, 2, 8, 9, 10, 16, 17, 18, 24, 25, and 26), ii/5 × 2 (MBTA administered on days 0, 1, 7, 8, 14, 15, 21, 22, 28, and 29), and iii/6 × 1 (MBTA administered on days 0, 6, 12, 18, 24, and 30) (Figure 4A). All regimens exhibited a comparable effect on tumor growth (Figure 4B). However, in terms of survival, the 5 × 2 regimen was the most effective, achieving an 87.5% survival rate (Figure 4C).

Within the 6 × 1 schedule, a personalized application mode was also evaluated, in which the injected volume of MBTA was adjusted according to the actual tumor size (MBTA volume set to half of the tumor volume). Compared with the standard fixed dose of 50 μl MBTA, this approach resulted a higher survival (50% vs. 37.5%); however, the difference did not reach statistical significance (Figure 4C). In subsequent experiments, this strategy was not pursued further, as administering higher doses of TLR agonizts was considered undesirable due to potential side effects.

### 2.4. Optimizing the dose size in MBTA immunotherapy of murine pancreatic adenocarcinoma

**Figure 5.**
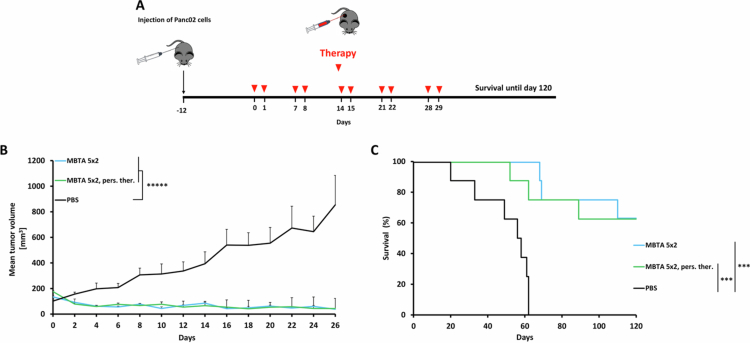
Optimization of immunotherapeutic dose size. **(A)** C57BL/6 mice were subcutaneously injected with Panc02 cells in the right flank. After 12 d, mice were randomized into three groups (*n* = 8/group) and treated by intratumoral application of MBTA according to the indicated regimens. **(B)** Tumor growth is shown as a growth curve. In the “MBTA 5 × 2, pers. ther.” group, the volume of mannan-BAM was personalized to match tumor size (mannan-BAM volume = 1/2 tumor volume). In the “MBTA 5 × 2” group 50 µl of standard MBTA was used. Statistical analysis was performed on AUC values using one-way ANOVA with Tukey's post hoc test (*****p < 0.0005). **(C)** Survival analysis is shown as a Kaplan–Meier curve, with log- rank test evaluation (*p < 0.05, **p < 0.01, ***p < 0.005, ****p < 0.001).

The personalized mode of MBTA application evaluated in the previous experiment with the 6 × 1 schedule did not improve survival. Furthermore, increasing MBTA doses in larger tumors could pose a risk of inflammatory side effects associated with TLR agonists.^9^ Consequently, in the next experiment, only the volume of mannan-BAM was adjusted according to tumor size (set to one-half of the tumor volume), while the other components were used at the previously established doses. The most effective therapeutic schedule, 5 × 2, was used when tumors reached a mean volume of 155.2 ± 55.9 mm^3^. Modified MBTA immunotherapy did not show any improvement compared with the standard regimen, either in terms of tumor growth inhibition or overall survival (Figure 5).

In addition, MBTA immunotherapy with half of the standard dose (25 μl) was tested; in this case, however, therapeutic efficacy was reduced (data not shown).

### 2.5. Immunotherapy in a murine model of colon adenocarcinoma

**Figure 6.**
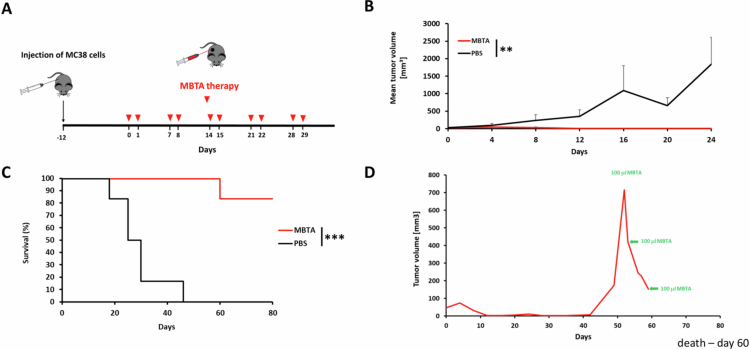
Immunotherapy of MC38 colon adenocarcinoma. (A) C57BL/6 mice were subcutaneously injected with MC38 cells in the right flank. After 12 d, mice were randomized into two groups (*n* = 6/group) and treated by intratumoral application of MBTA according to the indicated regimens. **(B)** Tumor growth is shown as a growth curve. Statistical analysis was performed on AUC values using one-way ANOVA with Tukey's post hoc test (**p < 0.01). **(C)** Survival analysis is shown as a Kaplan–Meier curve, log-rank test evaluation (***p < 0.005). **(D)** Attempt to cure tumor recurrence.

The aim of this experiment was to find out whether the acquired knowledge about the applicability of MBTA immunotherapy is also valid in the case of other tumors and their recurrences. The MC38 colon adenocarcinoma model was chosen. MC38 tumors are more immunogenic than Panc02. Treatment was administered according to the 5 × 2 schedule (Figure 6A) and resulted in a marked reduction of tumor burden (Figure 6B) accompanied by significantly prolonged survival (Figure 6C). The therapeutic results were comparable to the above results of pancreatic adenocarcinoma therapy. In one mouse a sudden recurrence of disease was observed. Additional MBTA treatment was applied, which led to a rapid regression of the tumor but was followed by the death of this animal (Figure 6D). We hypothesize that this outcome could be caused by tumor lysis syndrome, but this is just speculation that will require further study.

In addition, MBTA immunotherapy also proved effective in a murine model of recurrent MTT pheochromocytoma, where complete remission was achieved with personalized treatment (Fig. S1).

## 3. Discussion

The efficacy of intratumoral MBTA immunotherapy has been successfully verified in several cancer models.^6^ ^,^ ^7^ ^,^ ^10-13^ The promising results of MBTA immunotherapy were reflected in prolonged survival of experimental animals. Nevertheless, the optimal timing and dosing of therapeutics, the characterization of opsonization during MBTA therapy, strategies for treating tumor recurrences, and the use of personalized dosing remained largely unexplored. Therefore, this study focused on these aspects.

In the first experiment, we observed that inclusion of mannan-BAM substantially enhanced therapeutic efficacy when combined with the other components of the MBTA formulation, consistent with previously proposed roles of opsonization and phagocytosis in MBTA-mediated anti-tumor activity.^4-6^ The observation that no mice were cured by the combination of three TLR agonizts with an anti-CD40 antibody alone is unexpected and merits discussion. TLR agonizts exert a diverse anticancer effect, as demonstrated in many preclinical models and in human studies.^14^ A significant antitumor functional cooperation between resiquimod and poly(I:C) has been reported.^15^ The anticancer activity of agonistic anti-CD40 antibodies has also been described in numerous preclinical and clinical studies.^16^ ^,^ ^17^ Moreover, synergy between TLR agonizts and anti-CD40 antibodies in the induction of memory cells has been documented.^18^ In contrast to previously published data on the use of individual MBTA components (except mannan-BAM), whether administered alone or in combination, the failure of the mixture in our study may be explained by the extreme difficulty of treating pancreatic adenocarcinoma, whose unique features (e.g., dense stroma) contribute to its resistance. The most effective strategy in this model proved to be the simultaneous application of mannan-BAM, which activates the complement cascade, leading to opsonization, tumor cell killing, and phagocytosis. Mannan-BAM achieves artificial opsonization by anchoring mannan into the tumor cell membrane via the biocompatible anchor for membranes (BAM). Mannan-BAM is subsequently recognized by mannan-binding lectin (MBL), which triggers activation of the complement system and proteolytic cleavage of complement protein C3. Cleavage of C3 into C3a and C3b results in opsonization of tumor cells by iC3b.^4^ The strong synergistic effect of mannan-BAM with the other therapeutic components is consistent with the view of Underhill and Gantner^19^, who emphasized the synergy between signaling and phagocytic components in the innate immune response. Notably, mannan-BAM alone did not improve survival compared with PBS-treated controls. Although the relatively small group size may have contributed to biological variability, these findings further support the view that stimulation of phagocytosis alone is insufficient for effective tumor control and that the therapeutic efficacy of MBTA depends on the functional cooperation between phagocytic and signaling components.

Following recognition and activation of innate immunity, adaptive immunity is subsequently induced.^4^ ^,^ ^6^ This step is crucial for long-term survival, complete tumor eradication, and establishment of durable immune memory.^5^ ^,^ ^6^ Lymphocytic infiltration has been observed during MBTA immunotherapy in another difficult-to-treat cancer model, as reported by Uher et al.^10^ Based on to our previous findings, lymphocytes likely contribute not only to primary tumor elimination but also to the clearance of metastases.^6^ ^,^ ^8^ ^,^ ^10^

Overall, the first experiment confirmed the importance of opsonization and the strong efficacy of MBTA immunotherapy, the mechanism of which we have previously described in detail in a series of studies.^4^ The present study was not designed to directly investigate the cellular and molecular mechanisms underlying MBTA activity, and mechanistic interpretations are therefore based on previously published studies. One limitation of the first experiment is the unequal group sizes used in this experiment (e.g., *n* = 30 vs. *n* = 8). It may have affected the statistical power and robustness of comparisons and should therefore be considered when interpreting the findings.

Cancer recurrence is often fatal, most commonly due to metastatic disease. In preclinical research, resistance to recurrence is typically assessed by re-transplanting tumor cells into cured mice after a standard observation period (usually 100–120 d). In the second experiment, however, MBTA immunotherapy was applied to treat naturally occurring tumor relapses. Based on our experience, these mice would otherwise have succumbed rapidly to disease. Recurrence is generally characterized by extremely rapid tumor growth and death within days. Our aim was to determine whether treatment of tumor relapses could further enhance the efficacy of immunotherapy. This proved feasible in some cases, suggesting new treatment options for relapsed patients and underscoring the potential of a personalized MBTA immunotherapy approach.

To enhance the therapeutic effect while reducing the number of applications, potential side effects, and treatment costs, a 5 × 2 MBTA injection scheme proved to be the most advantageous while maintaining efficacy. One possible explanation for the limited benefit of the third injection cycle could be the development of tolerance to TLR agonizts, as proposed previously by Bourquin et al.^20^; however, this mechanism was not directly investigated in the present study. In any case, we emphasize the importance of administering immunotherapy over a sufficiently long period, thereby mimicking a vaccination schedule.^4^ Therapeutic doses must be sufficiently strong to elicit adequate signaling and immune responses. Increasing the overall dose of the immunotherapeutic mixture or its phagocytosis-stimulating component proved unnecessary, however, optimizing the ratio of MBTA components may further improve efficacy and will be addressed in future studies. Importantly, the MBTA dosage for treatment of tumor recurrence may need to be optimized based on tumor type. In one case, we observed a massive elimination of colon tumor tissue after MBTA injection, which was probably incompatible with life, possibly due to tumor lysis syndrome. Further investigation is required to clarify this mechanism.

Collectively, our results demonstrate that simultaneous targeting of signaling and phagocytic receptor ligands by MBTA immunotherapy is superior for the treatment of aggressive murine Panc02 pancreatic adenocarcinomas and MC38 colon adenocarcinomas, leading to a high rate of tumor eradication. Moreover, MBTA immunotherapy can successfully target recurrent tumors, as evidenced by complete cures or significant prolongation of survival in relapse-bearing mice. Nevertheless, management of recurrence immunotherapy is challenging and highly individualized. Whether future research will identify common therapeutic patterns or whether a fully personalized approach will always be required remains to be determined. Only extensive further studies will provide these answers.

While the subcutaneous models do not fully recapitulate the native tumor microenvironment, it provides a practical and widely used platform for initial evaluation of therapeutic efficacy and systemic anti-tumor immune responses.^21^ Further validation in orthotopic models will be important to confirm the translational relevance of these findings.

In conclusion, MBTA immunotherapy may represent a novel and effective strategy for treating cancers that are currently considered difficult to manage, particularly those with high rates of recurrence and metastasis.

## 4. Materials and methods

### 4.1. Materials

Mannan from *Saccharomyces cerevisiae* (Cat.No. M7504-1G) lipoteichoic acid (LTA) from *Bacillus subtilis* (Cat. No. L-3265-25MG), and polyinosinic:polycytidylic acid, sodium salt (poly(I:C)), Cat. No. P1530-25MG, were purchased from Sigma-Aldrich (St. Louis, MO, USA). Resiquimod (R-848), Cat. No. 4536/50, was obtained from Tocris Biosciences (Bristol, UK). BAM (Mw 4000), Cat. No. OT20B SUNBRIGHT OE-040CS 1 G, was purchased from NOF EUROPE (Grobbendonk, Belgium). The monoclonal antibody anti-CD40, Cat. No. BE0016-2 (rat IgG2a, clone FGK4.5/FGK45), was purchased from BioXCell (West Lebanon, NH, USA).

### 4.2. Cell lines and animals

The murine pancreatic adenocarcinoma cell line Panc02 (Cat. No. 300501) and the MC38 colon adenocarcinoma cell line (Cat. No. 305223) was purchased from Cytion (Heidelberg, Germany). Cells were maintained in Dulbecco’s modified Eagle medium (DMEM), Cat. No. LM-D1100/500, Biosera, Cholet, France, supplemented with 10% heat-inactivated fetal bovine serum (Cat.No. FB-1001/500, Biosera, Cholet, France) and antibiotics (Cat.No. P4333-100ML, Sigma-Aldrich, St. Louis, MO, USA). Cells were cultured at 37 °C in a humidified atmosphere containing 5% CO_2_. We use qPCR for mycoplasma testing. The DNA is isolated using the Zybio EXM 3000 with the Nucleic Acid Extraction kit, Zybio. Chongqing, China. We use GPO3-MGSO primers for detection.

Specific pathogen-free (SPF) C57BL/6JOlaHsd female mice (20g, 8 weeks old) were purchased from AnLab (Prague, Czech Republic). Mice were housed under SPF barrier conditions with free access to sterile food and water and maintained on a 12/12 h light-dark cycle.

### 4.3. Synthesis of mannan-BAM

Mannan-BAM was synthesized as previously described.^2^ Briefly, mannan was aminated by reductive amination^22^ and subsequently dialyzed overnight at 4 °C against PBS using dialysis tubing with a molecular weight cut-off of 3500 Da (Serva, Heidelberg, Germany). BAM containing an N-hydroxysuccinimide (NHS) reactive group was covalently bound to the amino groups of aminated mannan at pH 7.3 according to the protocol of Kato et al.^23^

### 4.4. Tumor transplantation

For subcutaneous transplantation, mice were subcutaneously injected with 4 × 10^5^ Panc02 or MC38 cells in 0.1 ml of DMEM without additives at a previously shaved right lower dorsal flank.

### 4.5. Treatment and its evaluation

*MBTA immunotherapy*: Once Panc02 or MC38 tumors were established (12 d after transplantation), immunotherapy was initiated. Mice received intratumoral injections of 50 µL of a therapeutic mixture containing 0.5 mg/mL R-848 (HCl form), 0.5 mg/mL poly(I:C), 0.5 mg/mL LTA, and 0.4 mg/mL anti-CD40, all dissolved in 0.2 mM mannan-BAM in PBS. Treatments were administered on days 0, 1, 2, 8, 9, 10, 16, 17, 18, 24, 25, and 26, or as otherwise indicated. Control mice received the same volume of PBS. To prevent potential leakage of the injected solution, tumors were covered with a thin layer of wound glue (Leukosan Adhesive, Tejpy, Prague, Czech Republic) before each application.

*TA immunotherapy*: Mice received the same therapeutic mixture as above but without mannan-BAM. Dosage, route of administration, and timing were identical to those used for MBTA immunotherapy.

*Man-BAM immunotherapy:* Mice received 0.2 mM mannan-BAM in PBS. Dosage, administration route, and timing were identical to those used for MBTA immunotherapy.

*Evaluation of treatment:* Tumor size was measured every other day using a calliper. Tumor volume (V) was calculated using the formula: V = (π/6)AB^2^, where A is the largest and B the smallest tumor dimension.^24^

### 4.6. Statistical analysis

The area under the curve (AUC) was calculated for comparisons across groups. AUC values were statistically analyzed using one-way ANOVA followed by Tukey’s *post hoc* test. Kaplan–Meier survival curves were compared using a log-rank test. Statistical analyzes were performed with STATISTICA 12 (StatSoft, Inc., Tulsa, OK, USA) and GraphPad Prism 8 for macOS (GraphPad Software, La Jolla, CA, USA). A *P*-value < 0.05 was considered statistically significant. For each analysis, the exact number of animals used is indicated in the figure legends. Data are presented as mean ± standard error of the mean (SEM).

The second experiment (recurrence therapy) was performed twice. Other experiments were run only once.

## Supplementary Material

Supplementary Material Supplementary File S1_Clean Version.docx

## Data Availability

The datasets generated during and/or analyzed during the current study are available from the corresponding author upon reasonable request.

## Abbreviations

BAM: biocompatible anchor for cell membrane
MBTA immunotherapy: mannan-BAM + TLR ligands (resiquimod, poly(I:C), lipoteichoic acid) + anti-CD40 antibody
TA immunotherapy: TLR ligands (resiquimod, poly(I:C), lipoteichoic acid) + anti-CD40 antibody
TLR: Toll-like receptor
LTA: lipoteichoic acid
