# Supplementary material for: Opsonization and timing as key determinants of MBTA immunotherapy efficacy in pancreatic adenocarcinoma and recurrence treatment
Source: Cancer Biol Ther. 2026 Jun 19;27(1):2683273. doi: 10.1080/15384047.2026.2683273 (PMC13285565; doi:10.1080/15384047.2026.2683273)
Supplement: Supplementary Material — Supplementary File S1_Clean Version.docx [file KCBT_A_2683273_SM2486.docx]

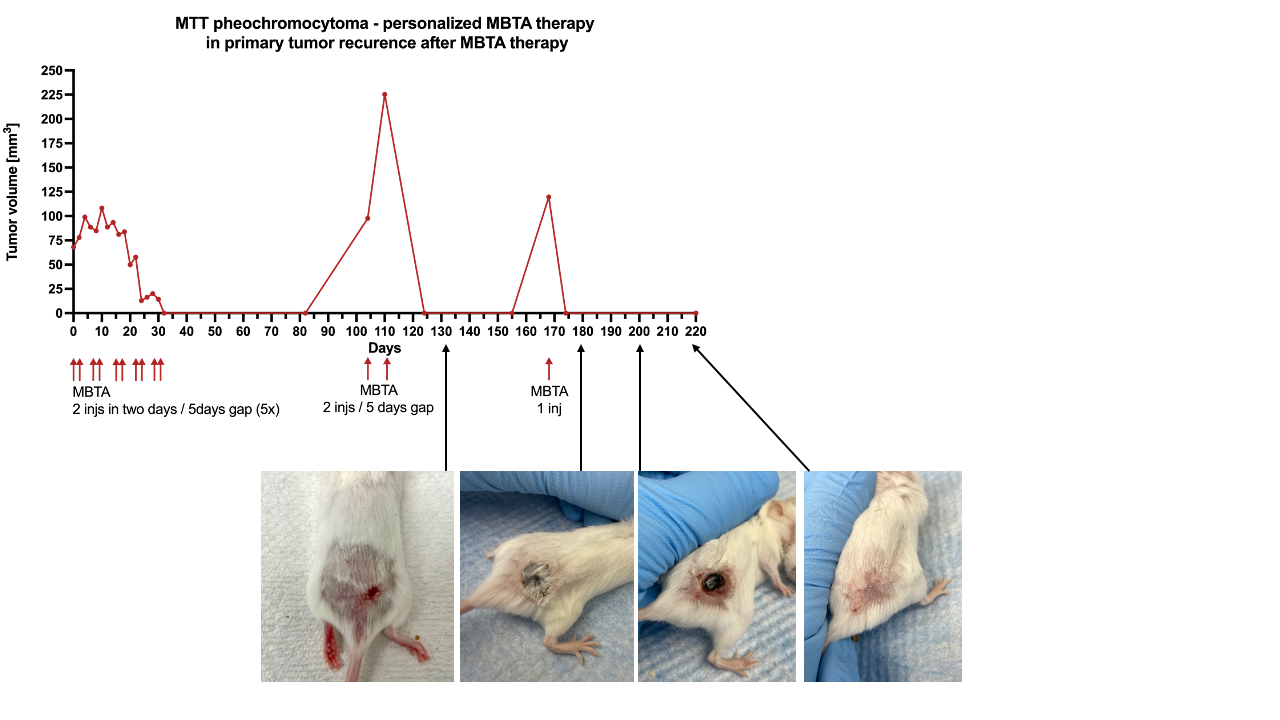


**S1** Primary tumor recurrence after intratumoral MBTA therapy in murine MTT pheochromocytoma model. After the development of subcutaneous MTT pheochromocytoma tumor (sc transplantation of 3 × 10^6^ MTT cells in 0.2mL, tumor volume 68.22mm^3^), the mice (n=10) were intratumorally treated with 50μl MBTA therapy consisted of five treatment cycles with five days gap (two MTBA injections in two days per one treatment cycle). The therapy resulted in tumor remission. However, after day 80 from the beginning of the therapy, the primary tumor of one mouse started growth. On days 104, the tumor volume was 97.64mm^3^. The same day 50μl of MBTA was injected. After six days, the tumor volume was 225.29 mm^3^ and the therapy was applied again. This two MBTA injections resulted in second remission for 30 days. However, then the second recurrence occurred, and tumor started growth again (day 168, tumor size 119.55 mm^3^). The same day, MBTA therapy was injected. This resulted in complete remission. The mouse was monitored for 100 days after the last injection with no tumor recurrence.
